# Nuclear Compartmentalization Contributes to Stage-Specific Gene Expression Control in *Trypanosoma cruzi*

**DOI:** 10.3389/fcell.2017.00008

**Published:** 2017-02-13

**Authors:** Lucía Pastro, Pablo Smircich, Andrés Di Paolo, Lorena Becco, María A. Duhagon, José Sotelo-Silveira, Beatriz Garat

**Affiliations:** ^1^Laboratorio de Interacciones Moleculares, Facultad de Ciencias, Universidad de la RepúblicaMontevideo, Uruguay; ^2^Departamento de Genética, Facultad de Medicina, Universidad de la RepúblicaMontevideo, Uruguay; ^3^Departamento de Genómica, Instituto de Investigaciones Biológicas Clemente EstableMontevideo, Uruguay

**Keywords:** gene expression regulation, RNA-Seq, subcellular fractionation, trypanosomes, differentiation, life cycle

## Abstract

In the protozoan parasite *Trypanosoma cruzi*, as in other trypanosomatids, transcription of protein coding genes occurs in a constitutive fashion, producing large polycistronic transcription units. These units are composed of non-functionally related genes which are pervasively processed to yield each mRNA. Therefore, post-transcriptional processes are crucial to regulate gene expression. Considering that nuclear compartmentalization could contribute to gene expression regulation, we comparatively studied the nuclear, cytoplasmic and whole cell transcriptomes of the non-infective epimastigote stage of *T. cruzi*, using RNA-Seq. We found that the cytoplasmic transcriptome tightly correlates with the whole cell transcriptome and both equally correlate with the proteome. Nonetheless, 1,200 transcripts showed differential abundance between the nuclear and cytoplasmic fractions. For the genes with transcript content augmented in the nucleus, significant structural and compositional differences were found. The analysis of the reported epimastigote translatome and proteome, revealed scarce ribosome footprints and encoded proteins for them. Ontology analyses unveiled that many of these genes are distinctive of other parasite life-cycle stages. Finally, the relocalization of transcript abundance in the metacyclic trypomastigote infective stage was confirmed for specific genes. While gene expression is strongly dependent on transcript steady-state level, we here highlight the importance of the distribution of transcripts abundance between compartments in *T. cruzi*. Particularly, we show that nuclear compartmentation is playing an active role in the developmental stage determination preventing off-stage expression.

## Introduction

Chagas' disease is caused by the protozoan parasite *Trypanosoma cruzi* (Kinetoplastida, Trypanosomatidae), which is transmitted to humans by diverse species of the Reduvidae bug family (Chagas, 1909). The infected insect releases the parasite metacyclic trypomastigote forms with its feces while feeding host blood, entering the host through the wound area. Within the host, the metacyclic trypomastigotes can invade nearby cells and therein differentiate into the intracellular amastigote forms. The latter multiply intracellularly and differentiate into bloodstream trypomastigotes, which are released into the circulation and infect cells from a variety of tissues, differentiating inside them into amastigotes. The bloodstream trypomastigotes can be ingested by bugs and transform in the vector's midgut into the replicative epimastigote and afterward, in the hindgut, into the infective metacyclic trypomastigotes.

Currently, there are approximately 6 to 8 millions of people suffering this disease and several millions are at risk of infection (WHO, 2015). Vector-borne transmission occurs in Central and South America but, since the parasite can also be transmitted by contaminated food, from mother to child and through contaminated blood or organ donations, Chagas' disease has spread to other continents (WHO, 2015).

Trypanosomatids show exceptional mechanisms for the expression of protein coding genes as the constitutive and polycistronic production of primary transcripts and mRNA maturation through the coordinate mechanisms of *trans*-splicing and polyadenylation. Since the mature messengers from the polycistronic transcriptional units (PTUs), are present in different levels, it is considered that post-transcriptional mechanisms should be actively controlling the gene expression in these organisms (Haile and Papadopoulou, 2007; Kramer and Carrington, 2011). Indeed, in *T. cruzi*, transcriptome studies have proved the existence of differences in mRNA abundance between genes which are consecutive in polycistronic transcriptional units (PTUs) (Minning et al., 2009; Smircich et al., 2015). These differences have been mainly attributed to mRNA stability and several *cis*-acting motifs and *trans*-acting factors have been identified (De Gaudenzi et al., 2005; Araujo and Teixeira, 2011; Bayer-Santos et al., 2012; Guerra-Slompo et al., 2012; Li et al., 2012). Besides, other post-transcriptional mechanisms occurring at the cytoplasm, such as those affecting mRNA localization (Cassola et al., 2007; Holetz et al., 2007; Cassola, 2011; Kramer, 2014) and translatability (da Silva Augusto et al., 2015; Smircich et al., 2015) provide additional steps for the control of mRNA availability for protein production.

The role of the nuclear compartment in the control of the cytoplasmic steady-state transcript levels has been recognized as crucial in eukaryotic gene expression regulation (Kohler and Hurt, 2007; Palazzo and Akef, 2012) and is also being studied in trypanosomatids (Nazer et al., 2011, 2012; Fadda et al., 2014; Antwi et al., 2016; De Gaudenzi et al., 2016; Kramer et al., 2016), for a very recent review see (Clayton, 2016). Although specific molecular actors involved in the nucleus-cytoplasmic transport in *T. cruzi* are being unraveled (Cassola and Frasch, 2009; Serpeloni et al., 2011a,b; Camara Mde et al., 2013; Inoue et al., 2014), no global analysis of the impact of nuclear compartmentation has been performed yet.

Transcriptome approaches in *T. cruzi*, have focused on the parasite life cycle, firstly studied by microarray analysis (Minning et al., 2009) and more recently, by RNA-Seq (Smircich et al., 2015; Houston-Ludlam et al., 2016; Li et al., 2016). In these cases, as for the majority of the transcriptome data from other organisms, the analyzed RNA is extracted from the whole cell disregarding the nuclear contribution to the total RNA population. This premise is supported by the selection of polyA tailed RNA. However, it has been claimed that eukaryotic gene expression analyses using whole cell lysates, inadvertently measure a substantial number of mRNAs that are restrained into the nucleus (Trask et al., 2009). More recently, this assertion has been endorsed by the demonstration of nuclear retention of spliced polyadenylated mRNA (Bahar Halpern et al., 2015).

Since the characterization of nuclear and cytoplasmic RNA may contribute to further understand trypanosomatid gene expression regulation, a comparative *in masse* analysis of nuclear and cytoplasmic RNA in *T. cruzi* epimastigotes was performed and the impact of nuclear-cytoplasmic RNA partitioning in whole cell RNA was evaluated. We found that the use of the cytoplasmic transcriptome does not significantly improve the estimation of protein abundance obtained from whole cell parasite transcriptome. However, we detected that up to 20% of the genes have differential transcript levels between nucleus and cytoplasmic compartments. Transcripts with higher level in the nucleus are significantly longer and have a higher GC content than the ones with higher content in the cytoplasm. Using data from the reported translatome and proteome for *T. cruzi* epimastigotes (de Godoy et al., 2012; Smircich et al., 2015), we observed that these transcripts are also characterized by scarce depiction of ribosome footprints and encoded proteins. In addition, we found that the transcripts enriched in the cytoplasmic fraction correspond to genes expressed in the epimastigote stage, while the ones enriched in the nucleus are distinctive of other life cycle stages. For selected specific transcripts, fluorescent *in situ* hybridization (FISH) was used to study the RNA subcellular localization in non-infective epimastigotes and in infective metacyclic trypomastigotes. Altogether, these results support an active role of nuclear compartmentalization in stage-specific gene regulation in *T. cruzi*.

## Materials and methods

### Parasites culture

The *T. cruzi* Dm28c clone (Contreras et al., 1988) was used. Epimastigotes were maintained at 28°C in liver infusion tryptose (LIT) medium supplemented with 10% heat inactivated fetal bovine serum (FBS). Metacyclic trypomastigotes were prepared as previously (Duhagon et al., 2009). Briefly, epimastigotes at stationary phase were incubated in TAU medium at 28°C for 2 h (Contreras et al., 1985). The parasites were then washed twice with PBS and immediately used.

### Parasite fractionation

To obtain nucleus and cytoplasmic fractions we used a previous reported methodology (Gomez et al., 1989) with a few modifications. Briefly, approximately 5 × 10^9^ parasites were used for each isolation procedure; the parasites were harvested at 1,700 g for 5 min. Cell pellets were resuspended in phosphate-buffered saline, washed twice, and resuspended in 3 volumes of ice-cold hypotonic buffer (10 mM HEPES, pH 7.9; 1.5 mM MgCl_2_; 10 mMKCl; 0.5 mM DTT; 1 mg/mL pepstatin; 0.5 mg/ mL leupeptin; 0.5 mM PMSF) and incubated on ice for 10 min. Nonidet P-40 was added to a final concentration of 0.2% and parasites were disrupted by 13 storks in a 15-mL glass Potter-Elvehjem Dounce homogenizer (Wheaton). Then sucrose was added to a final concentration of 0.35 M. An aliquot was separated and named “Whole cell” (Wc). The lysate was centrifuged at 500 g for 15 min at 4°C. The supernatant was separated and named “Cytoplasm” (C). The pellet was resuspended in 5 volumes of sucrose buffer (0.35 M sucrose; 10 mM HEPES, pH 7.9; 3.3 mM MgCl_2_; 10 mMKCl; 0.5 mM DTT; 1 mg/mL pepstatin; 0.5 mg/mL leupeptin; 0.5 mM PMSF) and centrifuged in a swinging bucket rotor at 1,100 g for 15 min at 4°C. The pellet was resuspended in the same sucrose solution and named “Nucleus” (N). Four independent replicates of the fractioning procedure were done.

### Western blot assay

Each Wc, C, and N protein fractions (corresponding to 5 × 10^6^ parasites per lane) were separated by electrophoresis in 10 or 12% SDS-PAGE and electro-transferred onto Hybond C Extra membranes (GE Healthcare) following standard procedures. Membranes were blocked by incubation in 5% skim milk powder in PBS-0.1% Tween and were then incubated for 1 h at room temperature with polyclonal, anti-TcTXN1 (Pineyro et al., 2011), anti-TcH2A, FioCruz, Brazil), anti-Tc38 polyclonal antibody (Duhagon et al., 2009), and anti-TcRBP40 (Guerra-Slompo et al., 2012), were used as purification controls. Bound antibodies were detected using an IRDye 800CW and 680CW Goat anti-Rabbit or anti-Mouse IgG (H + L) (Li-Cor), diluted 1:2,500 and analyzed in a G-Box (Syngene).

### RNA preparation and sequencing

RNA was isolated from the Wc, C, and N fractions using MirVana kit (Ambion), according to Total RNA purification manufacturer's protocol. The obtained RNA was treated with DNAse according to manufacturer's protocol (DNA-free, Ambion), quantified by Nanodrop (Thermo Scientific, USA) and Qubit 2.0 Fluorometer (Invitrogen) and its integrity was checked by Bioanalyzer (Agilent, USA). Genomic DNA control, in the cytoplasmic fraction, was done by qPCR against *gapdh* housekeeping gene. Then, RNA was treated with riboMinus (Invitrogen), the purified RNA was quantified by Nanodrop (Thermo Scientific, USA) and Qubit 2.0 Fluorometer (Invitrogen) and their integrity as well as rRNA depletion were analyzed by Bioanalyzer (Agilent, USA). Identical quantities of RNA derived from each of the four independent biological replicates were pooled to obtain the Whole cell, Nucleus and Cytoplasmic pooled samples. Pooled samples were sequenced by Illumina HiSeq2000 platform with a paired-end read running type and 100 bp cycle as a running condition at Macrogen (Korea).

### Data analysis

The obtained data were analyzed by CLC Genomics Workbench. In first place a 3′ quality trimming was done to the three sequence sets. To determine RNA transcript levels, the *T. cruzi* CLBrener Esmeraldo-like, genome release 4.2 from the TriTrypDB were used for RNA-Seq analysis included in the package. The reads per kilobase of transcript per million mapped reads (RPKM) were obtained for each gene in each condition. Only genes with 10 reads or more per transcript were used for quantification purposes.

Functional annotation of the NET and CET genes was performed using the Blast2GO tool (Conesa and Gotz, 2008) using a gene cut off of 10. Overrepresentation analyses were calculated by DAVID (Database for Annotation, Visualization and Integrated Discovery) (Dennis et al., 2003).

The intergenic RNA contribution for each fraction was calculated through the differences of reads that map to genome minus those that map to transcriptome (intergenic reads) as percentages.

For the fine location of the *trans*-splicing sites, we used SLaP mapper (Fiebig et al., 2014) *Trypanosoma cruzi* CLBrener Esmeraldo-like V6.0 was set as genome reference. Reads containing at least 8 nucleotides of the splice leader sequence were retrieved and the highest length upstream to the CDS or the one with more read counts was selected for each gene. Meanwhile, for the fine location of the polyadenylation sites, reads containing a track of at least 8 A were listed and the highest length downstream to the CDS or the one with more read counts was selected for each gene. To avoid inconsistencies of UTR delimitation due to partial maturation, only the data derived from the cytoplasmic transcriptome were used for these purposes.

GC-content (GC%) and GC-content in the third codon position (GC3) were calculated using in-house python scripts.

The RNAfold Vienna RNA Package 2.0 algorithm was used to calculate the minimum free energy for the 5′ and 3′ UTR of transcripts. Default parameters were used and the temperature was set at 37°C (Lorenz et al., 2011).

### Quantitative RT-PCR

cDNA was synthesized from 1 μg of DNAse treated RNA (DNA-free, Ambion) using Superscript III kit first strand synthesis (Invitrogen) and random hexamer primers. Quantification of specific products was done by qRT-PCR, using QuantiTect SYBR Green PCR Master Mix (Qiagen). Double stranded products were amplified using specific primers (Table S1) in a real time rotary analyzer RotorGene 6000 (Corbett). Relative amounts of target genes were calculated by normalization with the *gapdh* housekeeping gene that displayed similar values of absolute Ct in the quadruplicates: Wc (16.1 ± 0.2), N (16.1 ± 0.3) and C (16.5 ± 0.1) and of rpkm in the respective transcriptomes (216.5 for Wc, 263.6 for N and 266.5 for C). PCR reaction mixture containing 0.9 μM of each primer was carried out in a final volume of 10 μL. RNA levels were compared using the 2-ΔΔCT method (Livak and Schmittgen, 2001).

### Fluorescence *in situ* hybridization (FISH)

FISH analysis were done as previously described (Garcia-Silva et al., 2010) with some modifications. Briefly, epimastigotes were cultured, allowed to adhere to polylysine-coated microscope slides for 20 min at room temperature and, after washing twice in PBS, parasites were fixed with 4% paraformaldehyde in PBS for 10 min at room temperature, washed twice with PBS and further incubated in 25 mM NH_4_Cl for 10 min. Parasites were permeabilized with 0.2% Triton X-100 in PBS for 5 min. Slides were then blocked and prehybridized for 2 h at room temperature in bovine serum albumin 2%, 5 × Denhardt, 4 × SSC and 35% deionized formamide (hybridization solution). Assays were performed under denaturing conditions by heating slides at 75°C for 3 min just prior to the hybridization step (see Table S1 for used probes). Hybridization was performed overnight at 45°C in a humid chamber in the presence of 1 ng/ml of the indicated oligonucleotide conjugated either to fluorescein amidite (6-FAM) or to the cyanine 3 dye (Cy3) (Table S1). After hybridization, the slides were washed once in 2 × SSC plus 50% deionized formamide, once in 2 × SSC, once in 1 × SSC, and twice in 0.5 × SSC. Slides were mounted with Prolong (Molecular Probes) containing DAPI. Confocal images were acquired at room temperature using an OLYMPUS FV 300 with lasers Ar 488 and He-Ne 633 (Melles Griot). Merged images were obtained by superimposing the indicated images files in ImageJ software (Schneider et al., 2012).

### Availability of supporting data

The data sets supporting the results of this article are available in the Sequence Read Archive repository, BioProject ID: PRJNA342509.

## Results

### Transcriptomics of nuclear and cytoplasmic fractions of *T. cruzi* epimastigotes

**Figure 1:**
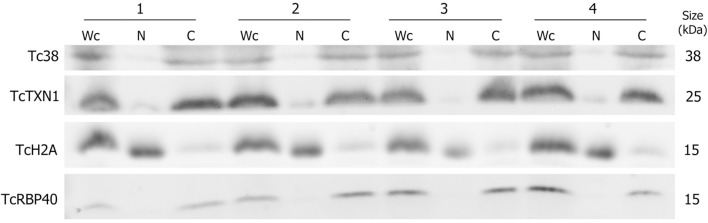
**Protein control of ***T. cruzi*** epimastigote subcellular fractions**. Western blot analysis of whole cell (Wc), nucleus (N) and cytoplasm (C) fractions using specific antibodies for selected protein markers: Tc38 (mitochondria), TcTXN1 (cytoplasm), TcH2A (nucleus), TcRBP40 (reservosome) are shown for four independent biological replicates (1 to 4). The molecular weights (kDa) of the proteins are indicated on the right.

In order to study the distinctive RNA contribution of the nuclear and cytoplasmic compartments to the total transcriptome of *T. cruzi* epimastigotes, a subcellular fractionation was performed. Proteins known to be specifically localized in: nucleus (histone TcH2A), cytoplasm (tryparedoxin TcTXN1), mitochondria (Tc38) and reservosomes (TcRBP40) were used in western blots to evaluate the purity of the fractions (Duhagon et al., 2009; Pineyro et al., 2011; Guerra-Slompo et al., 2012). The latter two markers were used to evaluate the frequent mitochondrial and reservosomal contamination of the nuclear fraction in this organism. As shown in Figure 1, the four independent replicates displayed very similar patterns. All the markers were present in the whole cell extract (Wc fraction), whereas the nuclear (N) fraction exhibited a conspicuous signal for the histone protein TcH2A and a faint to not detectable signals for the other three markers. On the contrary, the cytoplasmic (C) fraction showed strong bands for TcTXN1, Tc38, and TcRBP40, and a faint band for the nuclear marker.

**Figure 2:**
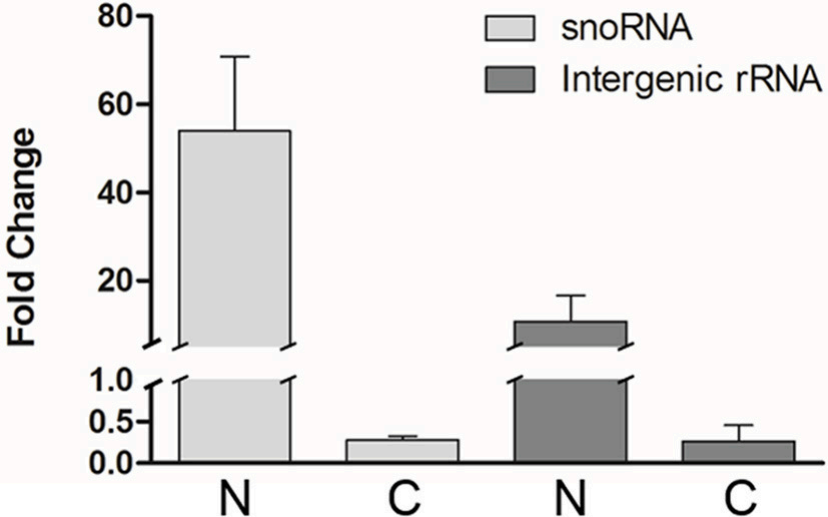
**Transcript control of ***T. cruzi*** epimastigote subcellular fractions**. Quantification of selected nuclear markers, snoRNA and intergenic rRNA was assessed by qRT-PCR relative to *gapdh* mRNA, in nuclear (N) and cytoplasmic (C) fractions of four independent biological replicates. Results are expressed as means and standard errors are indicated. (See Table S1 for gene ID and specific primers used).

Total RNA from each isolated fraction was extracted and qualitative and quantitatively analyzed (Figure S1). The four biological replicates yielded 0.6 ± 0.1 pg (mean ± S.E.) of total RNA per epimastigote cell. The nuclear RNA contribution represented 10 ± 1% of the RNA in the cytoplasm fraction per epimastigote cell. The differential distribution of well-established nuclear confined RNAs, such as unprocessed rRNA and snoRNA, was analyzed by quantitative RT-PCR (qRT-PCR). A remarkable enrichment of snoRNA and intergenic rRNA in the nuclear fractions was revealed (Figure 2). The small standard error among the four independent experiments argues in favor of the similarity of the quadruplicates (also supported by additional analyses presented in the following section). Besides, no genomic DNA could be detected by qPCR in the non-retrotranscribed RNA from the cytoplasmic fraction. Overall, the protein and RNA markers profile supported the adequacy of the nucleus and cytoplasmic fractions from the four independent replicates for a further RNA-Seq analysis.

Given the reproducibility of the replicates, the RNA extractions of the four biological replicates of each fraction were then pooled and the electrophoretic profile was analyzed. As observed for each individual experiment, the whole cell and the cytoplasmic pooled fractions displayed similar profiles whereas the nuclear fraction was more dissimilar (Figure S2). The pooled fractions were then sequenced as indicated in Materials and Methods section and the raw statistic data are shown in Table S2. RNA-Seq analysis using the *T. cruzi* CLBrener transcriptome yielded roughly 17 million mapped reads for Wc, N, and C. When the reads were mapped to the genomic sequences, we observed a higher proportion of mapped reads in the nuclear than in cytoplasmic fraction. This result was expected because of the presence of intergenic regions in the polycistronic RNA which are lost during maturation in the nucleus previous to the export to cytoplasm (Clayton, 2016). When the reads were mapped to the transcriptome sequences (see Data analysis in Materials and Methods section), we found that most of the 10,600 annotated transcripts present at least one mapped read: 9,079 in the Wc, 9,225 in the N and 9,011 in the C RNA fractions.

### Transcripts showing differential distribution between the nuclear and cytoplasmic fractions are found in *T. cruzi* epimastigotes

**Figure 3:**
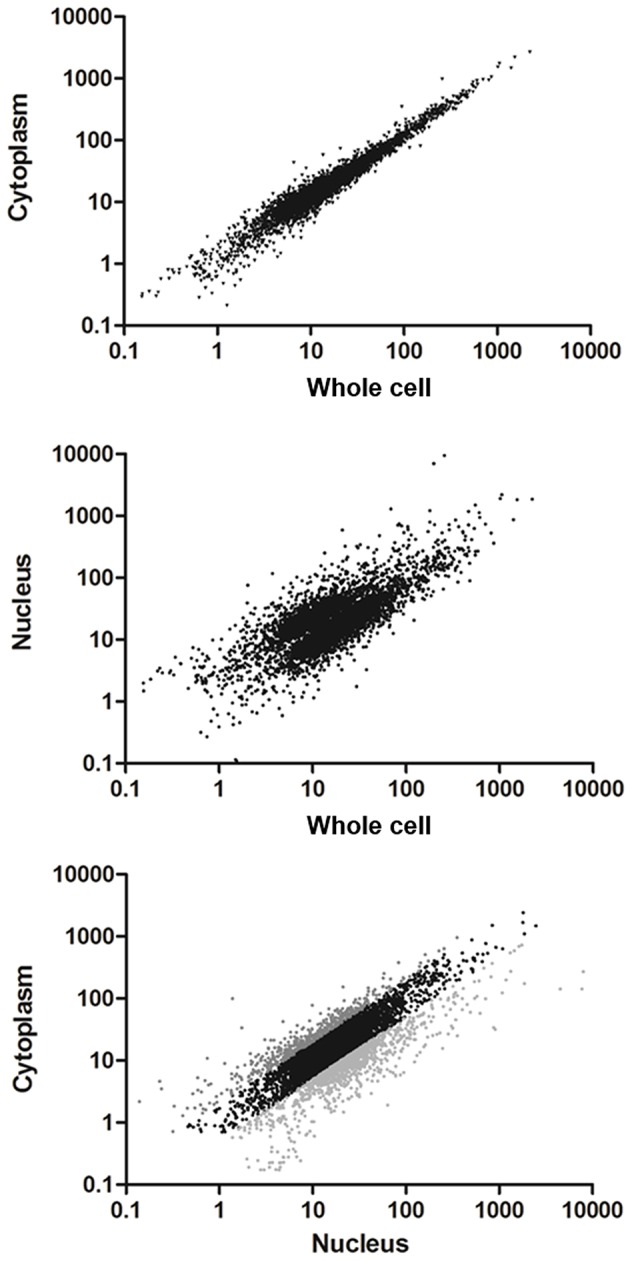
**Comparison of transcriptomes derived from whole cell to the subcellular fractionation of nucleus and cytoplasm in ***T. cruzi*** epimastigotes**. Scatter plot of the estimated expression levels as reads per kilobase of transcript per million mapped reads (RPKM) for genes with >10 reads in the transcriptomes. **Upper panel**: transcriptome derived from cytoplasm fraction vs. whole cell (Pearson correlation coefficient *r* = 0.98). **Middle panel**: transcriptome derived from nucleus fraction vs. whole cell (Pearson correlation coefficient *r* = 0.43). **Bottom panel**: transcriptome derived from cytoplasm vs. nucleus fractions (Pearson correlation coefficient *r* = 0.53). Differentially expressed genes (*FC* ≥ 2, *p* < 0.01) are shown in light gray for those whose transcript levels in cytoplasm were higher than in nucleus and dark gray for those whose transcript levels in nucleus were higher than in cytoplasm.

A great proportion of the annotated transcripts were present in the cytoplasmic and nuclear transcriptomes, indicating that the majority of the transcripts are exported to the cytoplasm. This finding is in agreement with the recent hypothesis of pervasive RNA maturation in *T. cruzi* (Smircich et al., 2015). However, since there might be differences between transcript abundance in the cell compartments, we quantitatively compared the transcriptomes. We found a strong correlation between C and Wc transcriptomes (Pearson correlation coefficient *r* = 0.98 *p* < 0.0001, Figure 3 upper panel). This indicates that the Wc transcriptome adequately represents the transcripts present in C, and therefore it is appropriate to describe the global mRNA steady-state level in this organism using whole cell transcriptome approaches. On the other hand, the nuclear transcriptome also correlated with the whole cell transcriptome, although with a smaller correlation coefficient (Pearson correlation coefficient *r* = 0.43, Figure 3 middle panel). Similarly, when we analyzed the correlation between the N and C transcriptomes we determined a Pearson correlation coefficient *r* = 0.53 (Figure 3 lower panel). These findings pointed us to the existence of transcript abundance differences between compartments. Consistent with these results on transcriptomes' correlations, similar correlations for Wc and C transcriptome with the epimastigote proteome (de Godoy et al., 2012) were deduced. As expected, a minor correlation coefficient with proteome was obtained for the nucleus transcriptome (Table S3).

In order to identify the sets of genes with differential transcript abundance between N and C, we compared their transcriptomes. A total of 1,182 genes (20% of the 6,039 genes with more than 10 reads in the Wc transcriptome) were found to have at least a 2-fold change between the two compartments (*FC* ≥ 2, gray colored in Figure 3 lower panel and Table S4 for the list of gene IDs). Among them, 444 were genes with cytoplasmic enriched transcript abundance (CET, dark gray in Figure 3 lower panel) and 738 had nuclear enriched transcript abundance (NET, light gray in Figure 3 lower panel), while 4,857 were not differentially distributed (NDT, black in Figure 3 lower panel). These results indicate that even when the transcriptomes from Wc and C are highly correlated, a set of genes show significant differences of transcript abundance between the nuclear and cytoplasmic compartments in *T. cruzi* epimastigotes.

**Figure 4:**
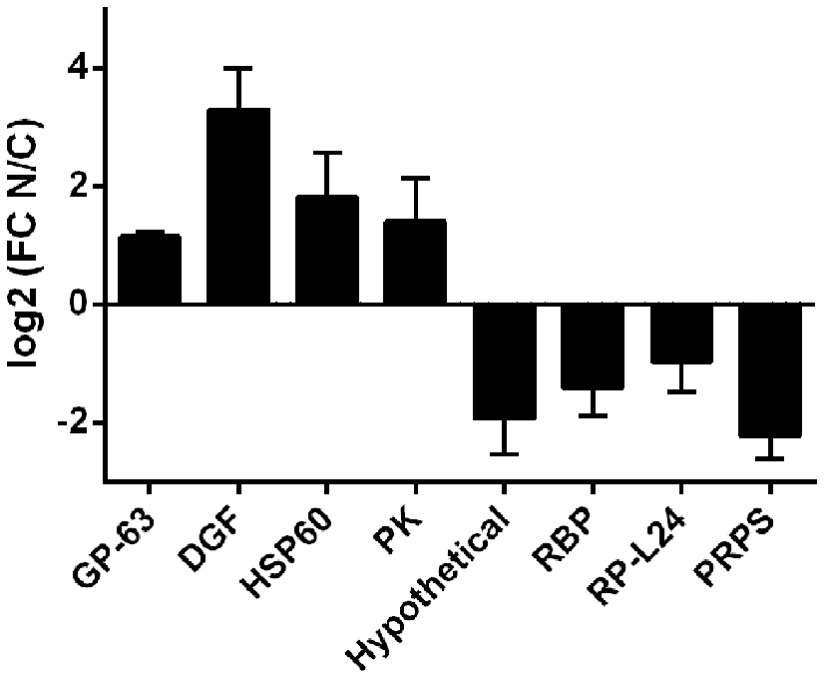
**qRT-PCR of selected transcripts in nucleus and cytoplasmic fractions in ***T. cruzi*** epimastigotes**. Transcript relative quantification was assessed by qRT-PCR in the four independent biological replicates. For normalization *gapdh* mRNA was used. Results are expressed as means of fold change between nuclear (N) and cytoplasmic (C) fractions. Standard errors are indicated. (GP-63, surface protease GP63, ID: TcCLB.506779.180; DGF, dispersed gene family protein 1, ID: TcCLB.509883.9; HSP60-Mit, chaperonin HSP60 mitochondrial, ID: TcCLB.507641.290; PK, protein kinase, ID: TcCLB.510343.15; Hypothetical, hypothetical protein, ID: TcCLB.506931.4; RBP, RNA-binding protein, ID: TcCLB.506649.80; RP-L44, ribosomal protein L24, ID: TcCLB.503611.20; PRPS, phosphoribosyl pyrophosphate synthetase, ID: TcCLB.508717.30). See Table S1 for specific primers used.

Robustness of the compartment distribution differences found by RNA-Seq was confirmed for eight genes by qRT-PCR in the four independent biological replicates (Figure 4). Furthermore, the high correlation of transcript quantification by qRT-PCR and by RNA-Seq (Pearson *r* = 0.86) strongly support the RNA-Seq results (Figure S3). These data together with the RNA markers (Figure 2) were used to compare the individual samples within each cellular fraction. The quadruplicates showed average correlations of *r* ≥ 0.96 with standard deviation ≤ 0.02 (Figure S4A). In addition, we found that the largest component of the variance of the samples, identified by PCA, comes from the identity of the cellular fraction (Figure S4B). These analyses validate the use of a pool of replicates of each subcellular fraction for the identification of differentially expressed genes.

### The genes with enriched transcript levels in the nucleus of *T. cruzi* epimastigotes have distinctive compositional and structural characteristics

We then investigated the association of properties such as mRNA length and base composition with the nuclear-cytoplasmic differential transcript distribution. Firstly, we had to identify the 5′ and 3′ ends for the UTRs, since in *T. cruzi* only CDS are annotated. We sought out the UTRs boundaries using the strategy outlined in Materials and Methods section which is based on the detection of at least 8 nucleotides of the splice leader for the 5′ UTRs and a track of at least 8 A for the 3′ UTRs. For the 1,182 genes exhibiting differentially distributed transcripts, the 5′ UTR and 3′ UTR could be assigned to 505 and 162 genes respectively. Nonetheless, complete transcripts could only be determined for 81 genes.

**Figure 5:**
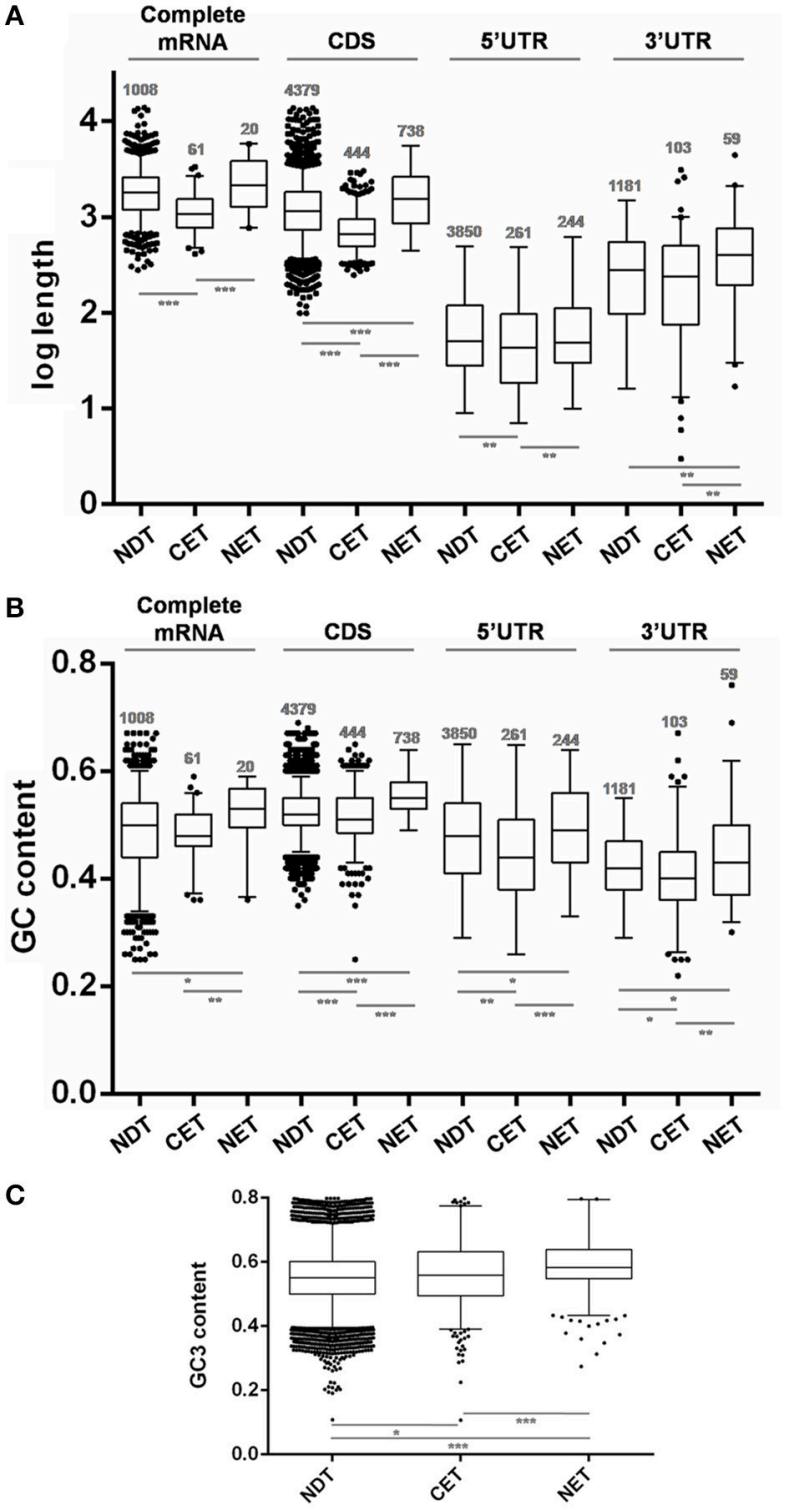
**Length and GC content of transcripts differentially distributed between nucleus and cytoplasm fractions of ***T. cruzi*** epimastigotes. (A)** Box plots of transcript gene length in nucleotides (nt). **(B)** GC content depicted as %. **(C)** GC3 content depicted as %. The analyses were done for the cytoplasmic enriched transcript (CET), nuclear enriched transcript (NET) and not differentially distributed transcript (NDT). The corresponding CDS, the 5′ UTR, the 3′ UTR and the complete mRNA (when both 5′ and 3′ UTR of a gene could be determined) are presented (^*^p < 0.01, ^**^p < 0.005, ^***^p < 0.0001).

The analysis of transcript length revealed that genes whose transcripts were enriched at the cytoplasm were significantly shorter than the non-differentially distributed transcripts (NDT) (*p* < 0.0001) while those whose transcripts were enriched at the nucleus were slightly longer than the ones in the cytoplasm (*p* < 0.0001) (Figure 5A). Although this difference mostly relied on CDS size a similar trend was observed for both UTRs.

Concerning the RNA composition, we observed that genes whose transcripts were enriched in the nucleus had a significantly higher GC% content than NDT genes (Figure 5B). This difference was seen in the GC content at CDS and a similar trend was observed at the UTRs. In addition, the CET GC content at the CDS and also at the determined UTRs was significantly lower than the NET and NDT. For the complete mRNA sequences, a similar tendency, with restricted significances probably due to the low number of determined sequences, was observed. A good agreement between the CDS GC content and GC3 usage was also found (Figure 5C). In addition, since the GC content could affect the RNA conformation, we also investigated the existence of significant thermodynamic differences at the determined UTRs of these sets of transcripts. A significant difference in the free energy was only found for the5′ UTRs of the NET genes when compared to the NDT genes (*p* < 0.01). The lower free energy of the 5′ UTRs of the NET genes may be predicting the presence of more stable structures at these regions (Figure S4).

These findings support that gene compositional and structural characteristics may underlay the differential transcript partitioning between nucleus and cytoplasm.

### The genes with enriched transcript levels in the nucleus of *T. cruzi* epimastigotes are poorly translated

**Figure 6:**
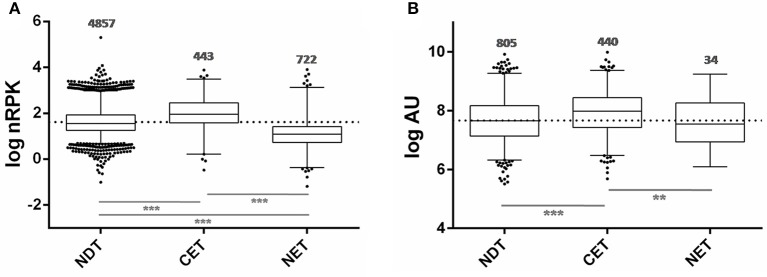
**Ribosome occupancy and Protein abundance of transcripts differentially distributed between nucleus and cytoplasm of ***T. cruzi*** epimastigotes. (A)** Box plots of ribosomal footprints from Smircich et al. (2015) as normalized reads per kilobase (nRPK) for genes with cytoplasmic enriched transcript abundance (CET), with nuclear enriched transcript abundance (NET) and with not differentially distributed transcript abundance (NDT). **(B)** Box plots of protein abundance for the indicated set of genes obtained from de Godoy et al. (2012) expressed in arbitrary units (AU) (^**^p < 0.005, ^***^p < 0.0001).

Taking advantage of the availability of data derived from ribosome profiling of *T. cruzi* epimastigote (Smircich et al., 2015), we determined the ribosome occupancy of the nucleus-cytoplasm differentially distributed transcripts. A high number of ribosome footprints were observed for CET, while NET had significantly fewer ribosome footprintings than either CET or NDT (Figure 6A). Using the quantitative proteomic data for *T. cruzi* epimastigotes (de Godoy et al., 2012) a similar result was obtained (Figure 6B). Interestingly, virtually all the CET genes were present in the proteome (99%, 440 out of the 444 - dark gray in Figure 3 lower panel), on the contrary, only a few NET genes are detected (6%, 44 out of the 738 - light gray in Figure 3 lower panel). In addition, while CET levels, measured in Wc transcriptome, significantly correlate with their proteomic expression, no correlation is seen for NET genes (Table S5). Consistently, the presence of transcripts of pseudogenes in the NET set was higher than expected by chance (122 out of 1,302 annotated pseudogenes, 36% increase, Fisher exact test *p* < 0.01). Even more, pseudogenes were underrepresented in the CET genes (21 pseudogenes, 62% decrease, Fisher exact test *p* < 0.01).

Taking together, these findings further support nuclear partitioning impact on protein content modulating transcript availability for translation.

### Off-stage transcripts are enriched in the nucleus compartment of the *T. cruzi* epimastigote stage

**Figure 7:**
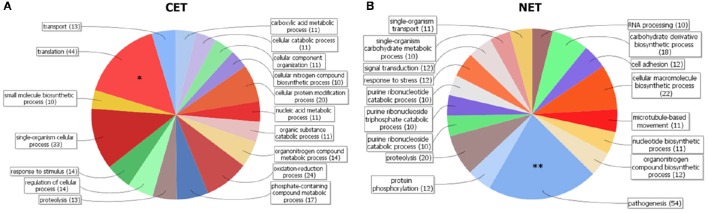
**Gene clustering of nuclear-cytoplasmic differentially distributed transcripts in ***T. cruzi*** epimastigotes**. Grouping of genes according to Blast2GO biological processes for genes with cytoplasmic enriched transcript (CET, **A**) and nuclear enriched transcript (NET, **B**) is shown. Note the differential increase of genes associated with pathogenesis in the NET group (marked with two asterisks). DAVID overrepresentation is indicated, [*p* value: 1e-10 (^*^) and 1e-6 (^**^)].

**Figure 8:**
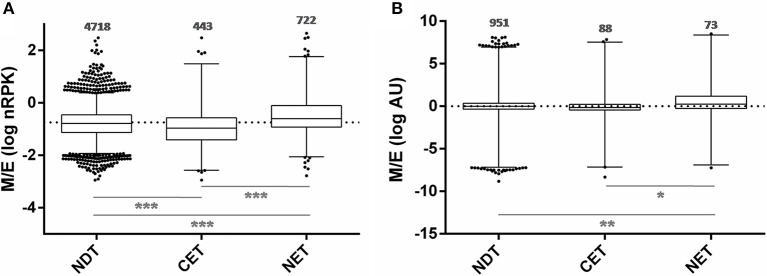
**Translatability and protein abundance of ***T. cruzi*** epimastigote nuclear-cytoplasmic differentially distributed transcripts in the metacyclic trypomastigote stage. (A)** Box plots of the metacyclic trypomastigote to epimastigote ratio of the ribosomal footprints from. Smircich et al. (2015) as normalized reads per kilobase (nRPK) for genes with cytoplasmic enriched transcript (CET), nuclear enriched transcript (NET) and not differentially distributed transcript (NDT) in *T. cruzi* epimastigotes. **(B)** Idem to **(A)** using the protein abundances expressed in arbitrary units (AU) from de Godoy et al. (2012) (^*^p < 0.01, ^**^p < 0.005, ^***^p < 0.0001).

In order to study the biological characteristics of the NET and CET genes, we performed an analysis of overrepresentation of gene ontology terms (Figure 7). Using the Blast2GO tool (Conesa and Gotz, 2008) we found that the CET genes are involved in biological processes of the replicative and non-infective epimastigote stage such as translation and metabolic pathways (Figure 7A). Conversely, genes encoding factors with active roles in life cycle stages different from the epimastigote stage, such as pathogenesis, have higher transcript abundance in the nucleus (Figure 7B). Concurrently, a detailed inspection of the NET genes exposed an overrepresentation of *trans*-sialidase, DGF, RHS, GP63 and MASP gene family's members (χ^2^, *p* < 0.001) and an underrepresentation of ribosomal proteins. The former families of genes have been extensively studied and their expression has been related to the infective metacyclic stage of the parasite (Cuevas et al., 2003; Bartholomeu et al., 2009; Kawashita et al., 2009; De Pablos and Osuna, 2012; Grynberg et al., 2012). Indeed, we found that the ribosome footprints of the epimastigote NET genes increase in the metacyclic trypomastigote stage while those of the epimastigote CET genes are diminished (Figure 8A). A similar pattern was obtained when comparing the metacyclic trypomastigote and epimastigote proteomic data from (de Godoy et al., 2012) (Figure 8B).

These findings support an active role of the nuclear compartment limiting the off-stage translation of genes from stages different from the actual that could occur due to the constitutive and massive characteristics of transcription in this parasite.

### Epimastigote nuclear-cytoplasmic transcript distribution changes after the transition to the metacyclic trypomastigote stage

**Figure 9:**
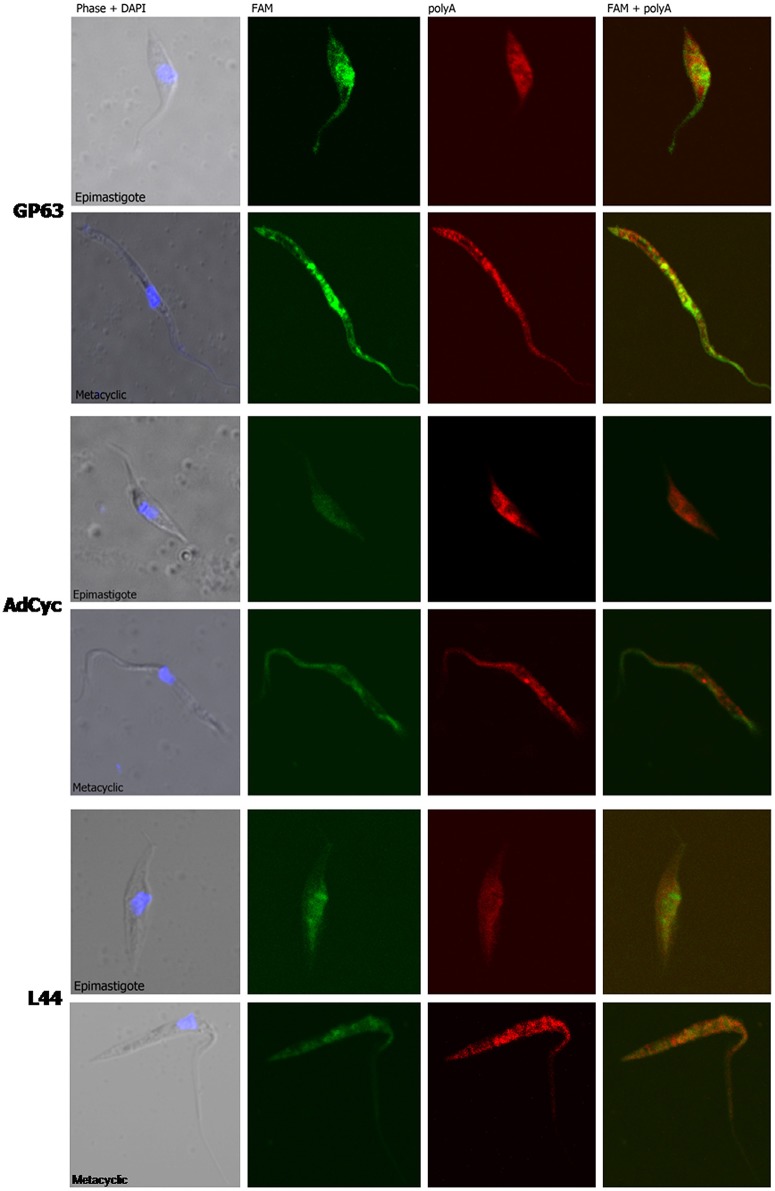
**mRNA localization in epimastigote and metacyclic trypomastigotes of ***T. cruzi*****. Confocal images of FISH assays of representative parasites using probes for: a surface protease GP63 transcript, ID: TcCLB.511211.90, GP63, upper panel. A receptor-type adenylate cyclase transcript, ID: TcCLB.511043.60, AdCyc, middle panel; and the 60S ribosomal protein L44 transcript, ID: TcCLB.507105.40, L44 bottom. At least 30 parasites were analyzed for each probe in each condition. Staining of epimastigotes (upper panels) and metacyclic trypomastigotes (bottom panels) are presented. These probes (See Table S2) were 5′ labeled with 6-FAM (green). For the detection of polyA tailed transcripts a 5′ Cy5 labeled polyT probe was used (red). DNA was visualized by DAPI staining (blue). Phase contrast images were obtained. Magnification 120x.

**Figure 10:**
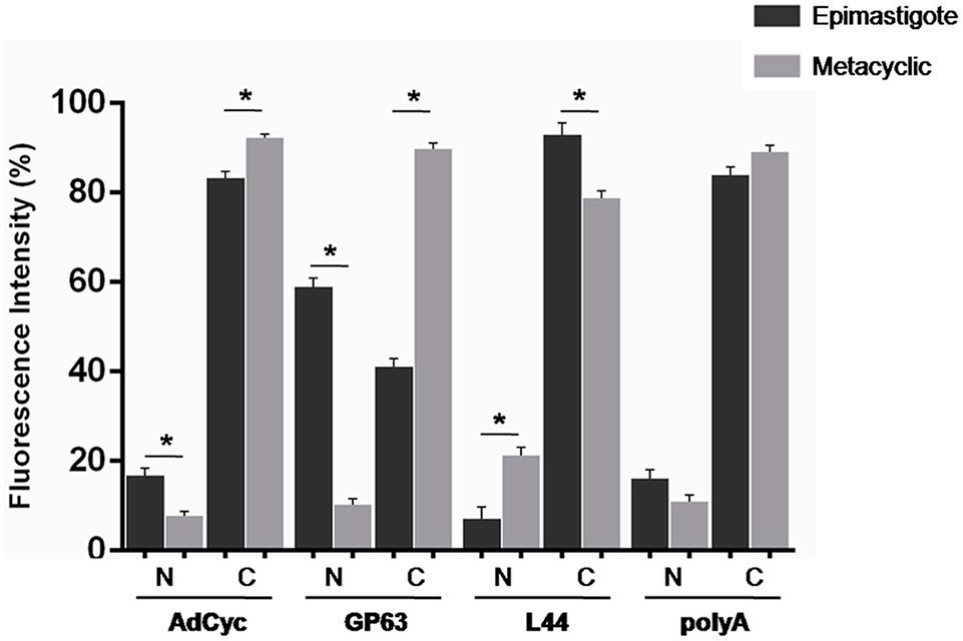
**Quantitative analysis of mRNA localization in epimastigote and metacyclic trypomastigotes of ***T. cruzi*****. Quantification of FISH assays of at least 30 parasites parasites using probes for: a receptor-type adenylate cyclase transcript, ID: TcCLB.511043.60, AdCyc, a surface protease GP63 transcript, ID: TcCLB.511211.90, GP63, the 60S ribosomal protein L44 transcript, ID: TcCLB.507105.40, L44, and polyA in epimastigotes (black) and metacyclic trypomastigotes (gray) (^*^p < 0.0001).

Taking into account all the above results, we hypothesized that transcript subcellular distribution is developmentally regulated. To test this hypothesis, we compared the localization of mRNAs belonging to CET (L44) and NET (GP63 and adenylate cyclase) in the non-infective epimastigote and the infective metacyclic trypomastigote, using fluorescent *in situ* hybridization (FISH). In contrast with the global mRNA localization, as seen by the detection of polyadenylated transcripts, these mRNAs showed developmental dependent localization (Figures 9, 10). The infection related gene, gp63, is faintly expressed in the epimastigote but increases its expression in the metacyclic trypomastigotes and amastigote stages (Cuevas et al., 2003). As shown in Figure 9, the gp63 transcripts were mainly localized in the nucleus in the epimastigote stage, while they acquired a cytoplasmic localization in the metacyclic trypomastigote stage. In addition, the superposition of polyA and the specific gp63 transcripts suggests an active translation in the metacyclic trypomastigote. A similar behavior was displayed by the adenylate cyclase transcript (also belonging to the NET gene set), being clear a depletion of the nuclear signal in the metacyclic trypomastigotes. For, the ribosomal protein L44 transcripts (belonging to the CET genes) a decrease of the colocalization with the polyA probe was observed in the transition from the epimastigote to the metacyclic trypomastigote stage. The quantitative analysis of the images for at least 30 parasites for each probe at the epimastigote or metacyclic trypomastigote stages, confirmed the displayed pattern changes (Figure 10).

These results support the existence of changes in the subcellular localization of transcript abundances accompanying the metacyclogenenesis developmental process in *T. cruzi*.

## Discussion

The steady-state level of proteins is the result of successive, alternative and combinatorial steps starting at transcription initiation. In *T. cruzi*, as well as in other trypanosomatids, the control of this step seems to be mostly absent. Therefore, trypanosomatids have been considered unique models for the study of post-transcriptional processes and their regulation. Indeed, processes like editing and *trans*-splicing were firstly described in trypanosomatids and later revealed to also occur, in other eukaryotes. However, while in eukaryotes the co-transcriptional RNA processing, surveillance and transport from nucleus to cytoplasm have been widely recognized as regulatory steps in gene expression (Kohler and Hurt, 2007), their survey in trypanosomes is very recent (Nazer et al., 2011, 2012; Fadda et al., 2014; Antwi et al., 2016; De Gaudenzi et al., 2016; Kramer et al., 2016). Likewise, genome-wide approaches have been used to identify the RNA nature and abundance distribution in the nucleus and the cytoplasm in many eukaryotes (Liao et al., 2010; Solnestam et al., 2012; Tilgner et al., 2012; Zaghlool et al., 2013; Bai et al., 2014), but such analyses have not yet been reported in trypanosomatids.

Seeking to study the role of the nuclear compartment in gene expression control in trypanosomes, we undertook cellular fractionation to obtain RNA from purified nucleus and cytoplasm fractions from *T. cruzi* epimastigotes to perform RNA-Seq and find whether transcripts were differentially distributed between these two compartments.

The fractionation reproducibility and the quality of the fractions were confirmed by protein and RNA compartment specific markers. An expected profile of protein markers was obtained for each of the independent experiment. The whole cell and cytoplasm extracts exhibited similar content of the different markers except for the nuclear marker TcH2A, whose almost disappearance in the later fractions supports the appropriateness of the selected fractionation method. Analogously, the mitochondrion, the reservosome as well as the cytoplasm markers exhibited weak to null signals in the nuclear fractions. The observed pattern may be pointing out to a negligible cross contamination of the fractions, however, the faint signal of TcH2A in the cytoplasm may also be attributed to the protein synthesis occurring at this compartment.

The content of total RNA per cell obtained from *T. cruzi* epimastigotes (0.6 ± 0.1 pg) is in good accordance with reported measurements in *T. brucei* bloodstream (0.5 pg, Haanstra et al., 2008, and procyclic forms 0.8 pg, Pays et al., 1993, and 1.1 pg, Haanstra et al., 2008). Variations of total RNA per cell content in the same range (0.5 to 1.6 pg) have also been reported for *Saccharomyces cerevisiae*, depending on the growth conditions (von der Haar, 2008). While the amount of total RNA in the nuclear fraction may vary with the physiological state of the cell, a rough estimation of 5 to 10% is commonly accepted based on the contribution of rRNA, mRNA and tRNA plus pre-mRNAs of 85, 2, and 13% respectively (Finka et al., 2015). Thus, we considered that the distribution of total RNA between nucleus and cytoplasm that we obtained (10 and 90% respectively) was consistent with the literature.

Thereafter, the RNA derived from the fractions of four independent experiments were pooled, sequenced and analyzed. Despite the fact that the genome of CLBrener Esmeraldo-like *T. cruzi* strain was used to map the RNA transcripts, the use of a paired end strategy strengthens the reliability of the assignments, and more than 9,000 genes (out of 10,600) were detected in each of the transcriptomes analyzed. Thus, in *T. cruzi* epimastigotes most of the genes are detected in the RNA extracted from whole cells or the nuclear or cytoplasmic fractions. While the good correlation between cytoplasmic and whole cell transcriptome (*r* = 0.98 *p* < 0.0001) was expected due to the reduced total RNA content of the nuclear fraction, the wide representation of transcribed genome in the cytoplasmic fraction (85%) further supports pervasive mechanisms for transcription and maturation as previously proposed (Smircich et al., 2015). However, though almost all the genes are shared by nuclear and cytoplasmic transcriptomes (84%), the poor correlation obtained between nucleus and cytoplasm transcripts levels (*r* = 0.53, *p* < 0.001) points out to the existence of an active compartment role. Indeed, 20% of the transcripts detected (NET+CET) showed at least a 2-fold change in expression levels between the two compartments analyzed. The reliability of these changes was confirmed for eight genes by qRT-PCR in each of the four independent biological replicates. A high correlation between qRT-PCR and RNA-Seq data was found. In addition, the low dispersion of the obtained qRT-PCR results provides evidence for the similarity of the used replicates. Altogether these findings support the existence of differentially distributed transcripts between nucleus and cytoplasm.

In agreement with previous reports for three human cell lines (Solnestam et al., 2012), where longer transcripts in the nucleus were inferred, we found that NET genes are significantly longer either than the NDT or CET genes. While this profile is mostly due to the size of the CDSs, the size of the UTRs also accompanies this trend. Concordantly, human genome-wide studies have revealed that highly expressed genes are significantly smaller and produce shorter mRNAs with shorter 3′ UTRs (Chiaromonte et al., 2003; Urrutia and Hurst, 2003). Similar results have been reported for *Arabidopsis thaliana* (Caldwell et al., 2010) and yeasts (Lackner et al., 2007; Lu et al., 2007). Recently, the length of mRNA has been related to developmental expression in *T. brucei* (Antwi et al., 2016). This finding is consistent with the role of nuclear compartmentalization in gene expression regulation during differentiation that we have here unraveled. Nonetheless, other features of gene sequences, such as G+C contents, are also associated with gene expression levels (Konu and Li, 2002). We found that NET genes have a significantly higher GC% content than NDT genes either at CDSs or UTRs. Since this trend is also accompanied by GC3 content at the CDS, codon usage may be contributing to further regulate gene expression of these transcripts. Codon usage has been identified as a major determinant of mRNA stability (Presnyak et al., 2015). In addition, high GC contents have been related to thermostability in different studies in bacteria (Nishio et al., 2003). Indeed, we found a significantly higher predicted thermodynamic stability for the 5′ UTR of NET genes which may be further influencing their low translation rate. Therefore, although the subcellular fractionation to obtain a cytoplasmic transcriptome may not introduce a significant advantage comparing to the whole cell transcriptome, we here provide evidence for a putative bias estimation due to length and composition of the transcripts in *T. cruzi* epimastigotes.

Considering that the transcriptome derived from a purified cytoplasmic fraction, following separation of the nuclear contribution, could better reflect the translationally active transcripts than the whole cell transcriptome, we analyzed the correlations between the transcriptome and the proteome. Again, we found that cytoplasmic transcriptome does not significantly improve the estimation of protein abundance obtained from whole cell parasite transcriptome. However, for the set of genes which have differential transcript abundance between the nucleus and cytoplasmic compartments, a functional bias was observed. The transcripts enriched in the cytoplasm correspond to genes which are: expressed in the epimastigote following ontology analyses; translationally active according to the ribosome footprint analysis (Smircich et al., 2015), and are widely represented in the epimastigote proteome (de Godoy et al., 2012). Meanwhile, an important number of the transcripts enriched in the nucleus correspond to pseudogenes. This is not surprising since several studies have demonstrated the functional roles of the pseudogene transcripts in post-transcription regulation mainly in the nucleus, either as competing endogenous RNAs or *trans*-acting RNA (Johnsson et al., 2014; Sen et al., 2014). A deeper analysis of the specific pseudogene transcript nature, sequence and structure could shed light to better understand their mechanism of action in this organism. In addition to pseudogenes, several *bona fide* gene transcripts showed a nuclear enrichment. From reported ribosome footprint studies, we found that these genes are less translated than the average in epimastigote. Likewise, very few of them are present in the epimastigote proteome. Ontology analyses revealed that the genes with nucleus enriched transcripts in the epimastigote are distinctive of other life cycle stages. Indeed, several genes belong to the extended multigenic families that codify for surface antigens such as the trans-sialidase (Schenkman et al., 1994; Nardy et al., 2016), the mucin associated surface proteins (Bartholomeu et al., 2009; dos Santos et al., 2012), the dispersed gene family (Kawashita et al., 2009; De Pablos and Osuna, 2012) and the glycoprotein family gp63 (Cuevas et al., 2003; Yao, 2010). Their function has been frequently associated with parasite protection and evasion of the host immune system (dos Santos et al., 2012; De Pablos et al., 2016; Houston-Ludlam et al., 2016). Since these gene families are absent in the phylogenetically closest free-living trypanosomatid *Bodo saltans*, it has been recently suggested that their acquisition could be related to the parasite adaptation to the hostile environments of the hosts (Jackson et al., 2016). Besides, the family of genes with hot spot for the insertion of retroelements, which also has nucleus enriched transcripts, is known to increase its expression upon exposure to γ radiation (Grynberg et al., 2012). Thus, the nuclear compartmentation may contribute to maintain the availability of transcripts necessaries to readily respond to environmental challenges.

Using the available data for ribosome footprinting and proteome of *T. cruzi* metacyclic trypomastigote, we analyzed the fate of the NET and CET epimastigote genes in the infective non-replicative parasite stage. We could demonstrate a significant change of the ribosome occupancy of transcripts and encoded proteins from these two transcript sets during metacyclogenesis, that is in good agreement with a role of the nuclear compartment in determining which mRNAs will be retained or released to the translationally active cytoplasmic compartment.

We further studied whether this developmental relocalization could be also proved through direct visualization of selected transcripts. In epimastigotes, the ribosomal protein L44 mRNA is found in the cytoplasm, mainly at the nucleus boundaries, and colocalizing with the polyadenylated mRNAs. Meanwhile, in addition to its reported decreased expression in the metacyclic trypomastigote, a wider spread pattern along the parasite, with no nuclear delimitation and a lesser colocalization with polyadenylated mRNAs was observed. On the other hand, the adenylate cyclase, known to be expressed in the metacyclic trypomastigote stage showed a diminished nuclear transcript content in this stage in comparison to the epimastigote stage. The change of location was even more evident for a gp63 gene involved in the infectivity process, whose transcript was markedly restricted to the nuclear translational inactive compartment in the non-infective epimastigote stage, moving to the translationally active cytoplasm, with a good co-localization with polyadenylated mRNAs, in the infective metacyclic trypomastigote stage.

These findings support an active role of the nuclear compartment confining the transcripts that are not immediately needed to produce proteins but may be rapidly required upon differentiation. The analysis of the actual contribution of each transcript species, i.e., unmature, partial or complete processed transcript to the nuclear content would shed light about the implied regulatory mechanisms. In addition, whether this differential RNA distribution is achieved by nuclear retention or differential nuclear-cytoplasm RNA decay should be investigated. Altogether these results provide strong evidence indicating that the nucleus–cytoplasm partitioning constitutes a control step that contributes to the differential regulation of a life cycle specific set of genes in *T. cruzi*.

## Author contributions

Conceived and designed the experiments: MD, JS, and BG; Performed the experiments: LP; Analyzed the data: LP, PS, AD, LB, MD, JS, and BG; Contributed reagents/materials/analysis tools: PS, AD, LB, JS, and BG; Wrote the paper: LP, PS, MD, JS, and BG.

## Funding

This work was supported by CSIC and CAP UdelaR, ANII and PEDECIBA, Uruguay.

### Conflict of interest statement

The authors declare that the research was conducted in the absence of any commercial or financial relationships that could be construed as a potential conflict of interest.
