# Supplementary material for: Nuclear Compartmentalization Contributes to Stage-Specific Gene Expression Control in Trypanosoma cruzi
Source: Front Cell Dev Biol. 2017 Feb 13;5:8. doi: 10.3389/fcell.2017.00008 (PMC5303743; doi:10.3389/fcell.2017.00008)
Supplement: Supplementary file 1 [file Table1.PDF]

Table S1. Specific primers used

	Target Gene CL Brener Esmeraldo like	Sequence primer Forward or probe	Sequence primer Reverse
**snoRNA**	TcCLB.510283.26	5'- ATGATGATTGACGTAACATCACAGAC -3'	5'- TCAGACTGGGGCGACAGTGTTTC -3'
**Gapdh**	TcCLB.506943.50 - TcCLB.506943.60	5'- CGACAACGAGTGGGGATACT -3'	5'-CTACAACCTTGCCGAACGAT -3'
**rRNA-Lgama-Lalfa**		5'- aaggggcaacagagaacctggg -3'	5'- agctcaccgtgggaggattg -3'
**rRNA-Lgama-Lbeta**		5'- gtcattgttgtgagttctgcgc -3'	5'- ctctatgtaccagagcattgtatgc -3'
**PK**	TcCLB.510343.15	GTTGAGGCCTTCCTGCTTGT	CAAATGCGTTTCTGTCATTGC
**GP63**	TcCLB.506779.180	CTGCTTGACTGGGGAGAACC	CACACTTAGCGTGCCCTCAC
**DGF-1**	TcCLB.509883.9	5'- GACGGTGTTGGACTGTGGTG -3'	5'- ACTCACAGCCGCTGATGCTC -3'
**HSP60-Mit**	TcCLB.507641.290	5'- GTGCAGGAGGGCATTGTTC -3'	5'- GTGAGTGACGAGTCGCCAAG -3'
**Hypothetical**	TcCLB.506931.4	5'- TGGGTAGCCTCACGGAAAGT -3'	5'- TGCGTCCATTGGTAGAATGC -3'
**RBP**	TcCLB.506649.80	5'- TGTGGTTTTGCCCAAGAATG -3'	5'- GCTGTCAAACTGCACCAAGG -3'
**RP-L24**	TcCLB.503611.20	5'- AAGAAGAACCCCCGCTTTGT -3'	5'- GGTTGTCATCTTGCGGTTGA -3'
**PRPS**	TcCLB.508717.30	5'- GGTGGATGCGTTGGTAGAGG -3'	5'- ACCGGGATATGCAACACTCC -3'
**polyApolM**	TcCLB.510317.30	5'- TAGCGATCTCGATATTGTGCTG -3'	5'- GCAGCAGCGGAGGGAACTC -3'
**polyApol_Inm**	TcCLB.510317.34	5'- GGTGCCTCCGTTGCAGATAG -3'	5'- ggggattagagaggggttgg -3'
**L44_FAM**	TcCLB.507105.40	5'- AATGATGGACTTGCAGTTGGAGCATTGGAGCTTCAGGACA -3'	
**GP63_FAM**	TcCLB.511211.90	5'- AGACGACCATCATCCAGCCGAACGCACGGACCCGCCCA -3'	
**AdCyc_FAM**	TcCLB.511043.60	5'- GCGACGAGCACGGACAGTAATAATCGAATCATCCGTCGA -3'	
**polyT-Cy5**		5'- TTTTTTTTTTTTTTTTTTTTTTTTTTTTTT -3'	
