# Supplementary material for: Nuclear Compartmentalization Contributes to Stage-Specific Gene Expression Control in Trypanosoma cruzi
Source: Front Cell Dev Biol. 2017 Feb 13;5:8. doi: 10.3389/fcell.2017.00008 (PMC5303743; doi:10.3389/fcell.2017.00008)
Supplement: Supplementary file 2 [file Table2.PDF]

Table S2. RNAseq data of subcellular fractions of *T. cruzi* epimastigotes

	Whole cell	Nucleus	Cytoplasm
**RNA-seq data (nt)**	9,727,558,056	7,310,872,476	9,360,517,996
**Average length (nt)**	100	100	100
**Mean library size (nt)**	450	409	438
**Reads mapped to genome**	23,830,921	23,460,632	21,076,033
**Reads mapped to transcriptome**	18,739,614	17,793,314	16,532,099
**Reads mapped to annotated CDSs**	6,316,999	8,344,884	4,907,619
