# Supplementary material for: Nuclear Compartmentalization Contributes to Stage-Specific Gene Expression Control in Trypanosoma cruzi
Source: Front Cell Dev Biol. 2017 Feb 13;5:8. doi: 10.3389/fcell.2017.00008 (PMC5303743; doi:10.3389/fcell.2017.00008)
Supplement: Supplementary file 3 [file Table3.PDF]

Table S3. Correlation analysis between protein abundances and transcriptomes from whole cell, cytoplasmic and nuclear fractions of *T. cruzi* epimastigotes

Fraction / Set	Transcriptome*	Proteome**	Spearman	Significance
**Whole cell**	6039	1280	0.5494	<0.0001
**Cytoplasm**	7746	1280	0.5638	<0.0001
**Nucleus**	8276	1280	0.4231	<0.0001

* Number of genes with more than 10 reads per transcript in the corresponding fraction or set.

** Number of transcripts for each transcriptome set whose encoded proteins were quantitatively determined by de Godoy et al. .
