# Supplementary material for: Nuclear Compartmentalization Contributes to Stage-Specific Gene Expression Control in Trypanosoma cruzi
Source: Front Cell Dev Biol. 2017 Feb 13;5:8. doi: 10.3389/fcell.2017.00008 (PMC5303743; doi:10.3389/fcell.2017.00008)
Supplement: Supplementary file 5 [file Table5.docx]

**Table S5. Correlation analysis between protein abundances and transcripts enriched in the cytoplasmic and nuclear fractions of *T. cruzi* epimastigotes**

| **Fraction / Set** | **Transcriptome*** | **Proteome**** | **Spearman** | **Significance** |
| --- | --- | --- | --- | --- |
| **CET** | 444 | 440 | 0.5847 | <0.0001 |
| **NET** | 738 | 44 | 0.2327 | 0.1854 |

* Number of genes with more than 10 reads per transcript in the corresponding fraction or set

** Number of transcripts for each transcriptome set whose encoded proteins were quantitatively determined by de Godoy *et al*. {de Godoy, 2012 #805}.
