# Supplementary material for: Nuclear Compartmentalization Contributes to Stage-Specific Gene Expression Control in Trypanosoma cruzi
Source: Front Cell Dev Biol. 2017 Feb 13;5:8. doi: 10.3389/fcell.2017.00008 (PMC5303743; doi:10.3389/fcell.2017.00008)
Supplement: Supplementary file 6 [file Presentation1.pdf]

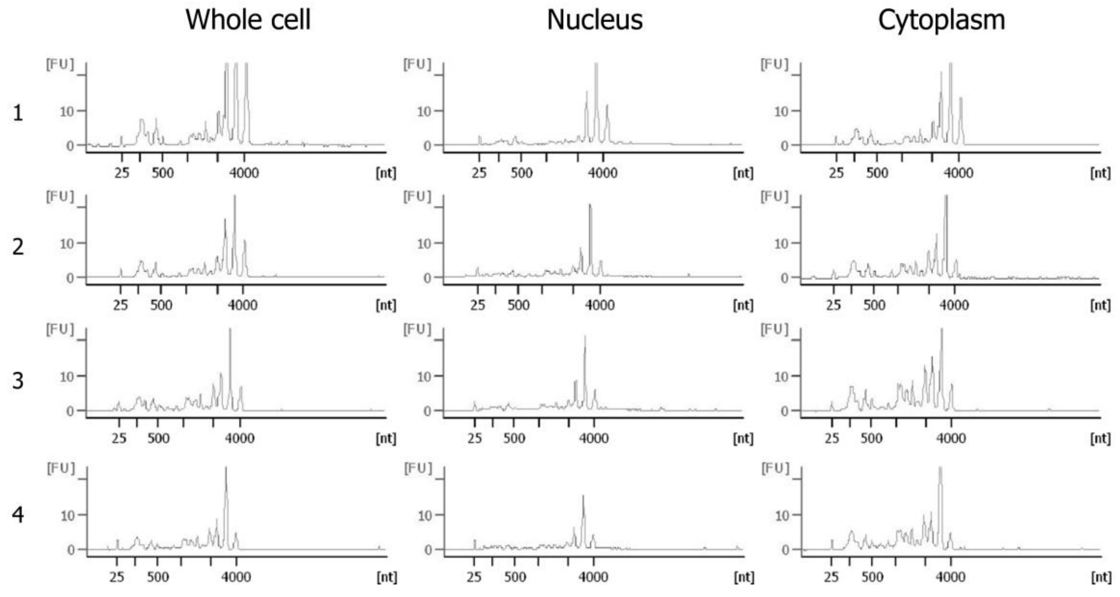

Figure S1. RNA electrophoretic profile of subcellular fractions of *T. cruzi* epimastigotes

The RNA electrophoretic profiles of whole cell, nucleus and cytoplasm fractions of four independent biological replicates are shown (1 to 4).

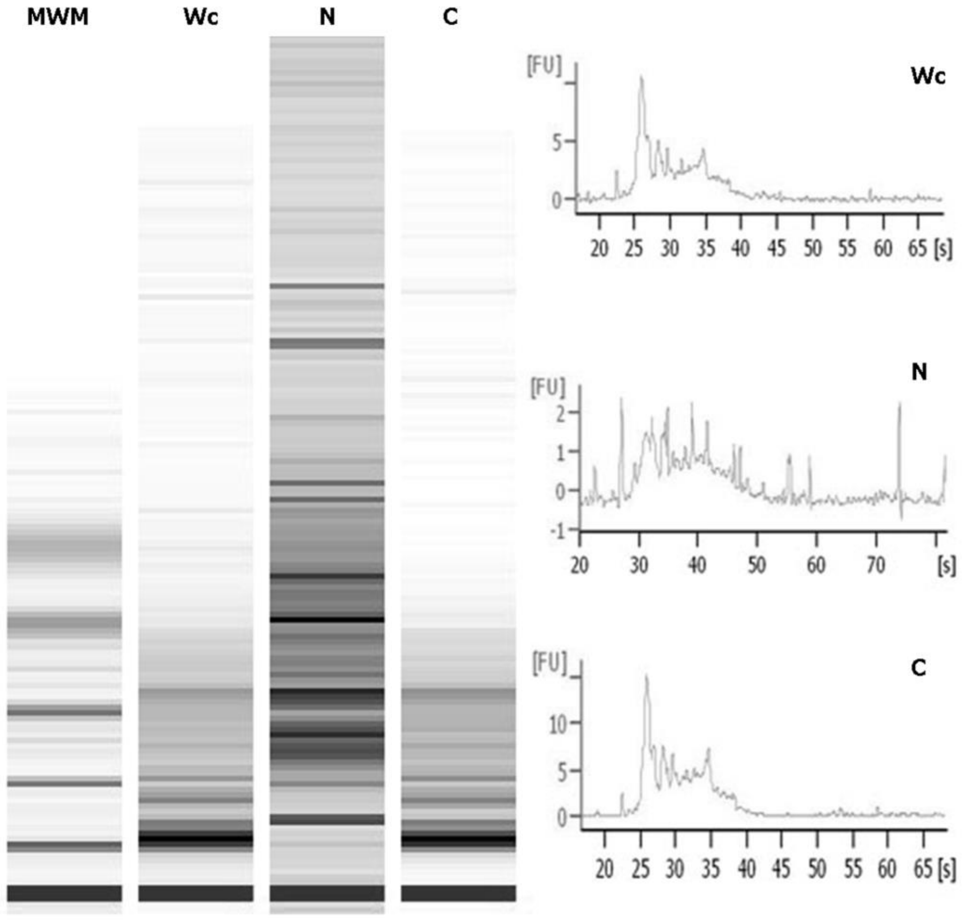

Figure S2. RNA electrophoretic profile of pooled subcellular fractions of *T. cruzi* epimastigotes

The RNA electrophoretic profile for the pooled whole cell (Wc), nucleus (N) and cytoplasm (C) fractions of four independent biological replicates is shown. MWM: molecular weight marker.

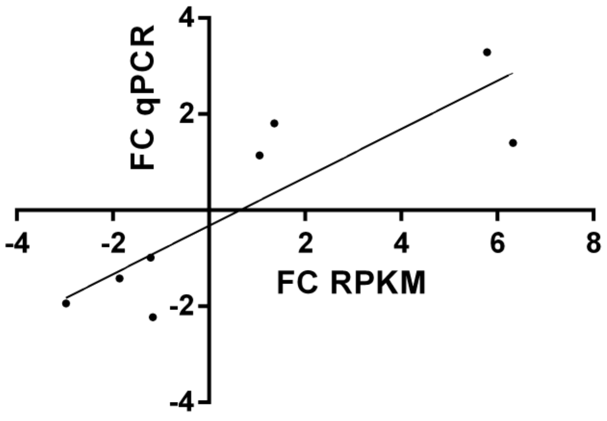

Figure S3. Correlations between qRT-PCR and RNA-Seq results
FC of RPKM in N / C *vs* FC of qRT-PCR in N / C.

A

	Wc1	Wc2	Wc3	Wc4
Wc1	1.00	0.95	0.98	0.95
Wc2		1.00	0.96	0.98
Wc3			1.00	0.98
Wc4				1.00

**Mean**	**0.97**
**SD**	**0.02**

	N1	N2	N3	N4
N1	1.00	0.94	0.97	0.96
N2		1.00	0.95	0.98
N3			1.00	0.96
N4				1.00

**Mean**	**0.96**
**SD**	**0.02**

	C1	C2	C3	C4
C1	1.00	0.96	0.98	0.96
C2		1.00	0.96	0.98
C3			1.00	0.96
C4				1.00

**Mean**	**0.97**
**SD**	**0.01**

B

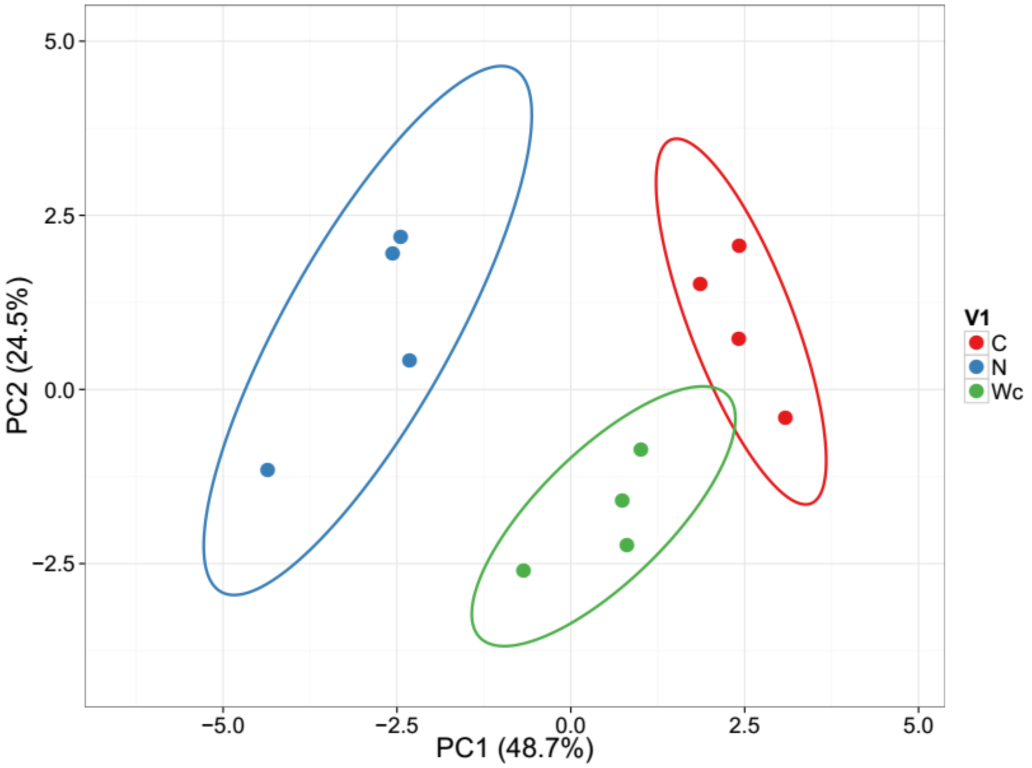

Figure S4. Analysis of the expression patterns on the independent quadruplicate fractions that were pooled for RNA-Seq

The quantification of transcripts of 12 genes (see Table S1 for IDs and primers) was done by qRT-PCR on each sample: whole cell (Wc), nucleus (N) and cytoplasm (C) fractions of four independent biological replicates. For normalization *gapdh* mRNA was used. **(A)** Pearson correlation analysis **(B)** Principal component analysis plot.

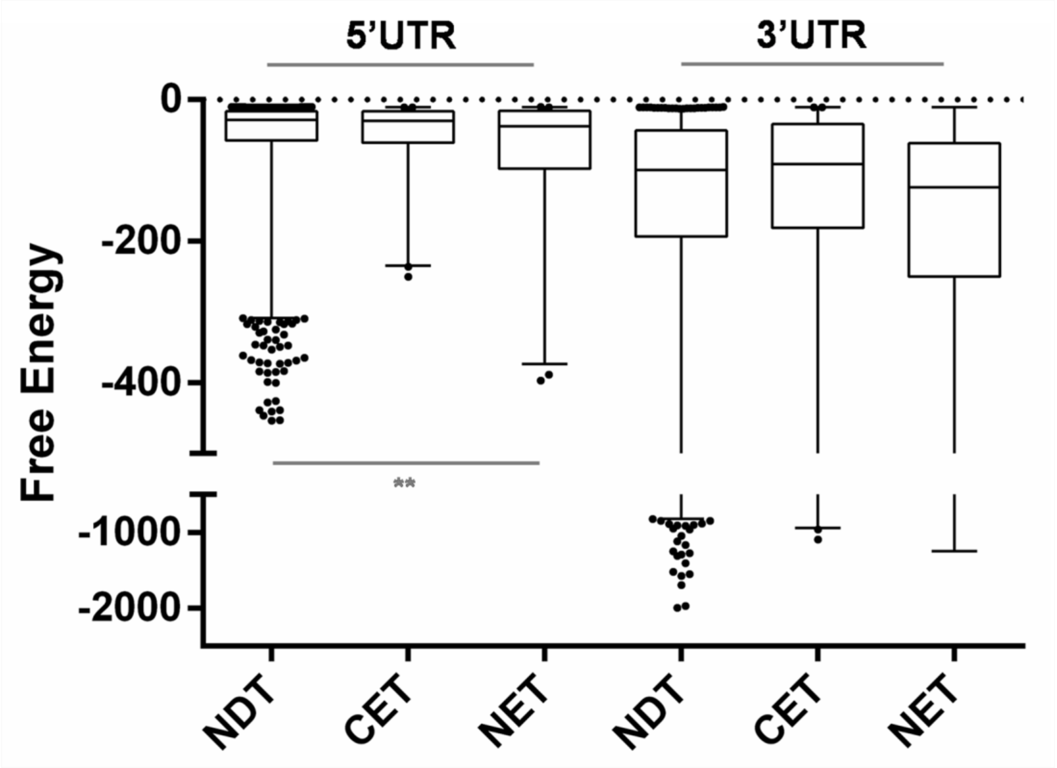

Figure S5. Free energy for the UTR predicted structures of genes with differentially distributed nucleus-cytoplasm abundance transcripts in *T. cruzi* epimastigotes
Box plots of the free energy (kcal/mol) for the predicted structures both at the 5'UTR and 3'UTR of genes with cytoplasmic enriched transcript abundance (CET), with nuclear enriched transcript abundance (NET) and with not differentially distributed transcript abundance (NDT) (** test t p<0.01).
